# Manipulation of Metabolic Pathways and Its Consequences for Anti-Tumor Immunity: A Clinical Perspective

**DOI:** 10.3390/ijms21114030

**Published:** 2020-06-04

**Authors:** Huang-Yu Yang, Chao-Yi Wu, Jonathan D. Powell, Kun-Lin Lu

**Affiliations:** 1 Kidney Research Institute, Department of Nephrology, Chang Gung Memorial Hospital, College of Medicine, Chang Gung University, Taoyuan 333, Taiwan; 2 Division of Allergy, Asthma, and Rheumatology, Department of Pediatrics, Chang Gung Memorial Hospital, College of Medicine, Chang Gung University, Taoyuan 333, Taiwan; joywucgu@hotmail.com; 3 Bloomberg~Kimmel Institute for Cancer Immunotherapy, Sidney Kimmel Comprehensive Cancer Center, Department of Oncology, Johns Hopkins University School of Medicine, Baltimore, MD 21287, USA; 4 Department of Medical Education, Chang Gung Memorial Hospital at Linkou, Taoyuan 333, Taiwan

**Keywords:** metabolic pathway, immunometabolism, immune checkpoint inhibitors, tumor microenvironment, cancer, anti-tumor treatment, glutamine, metformin

## Abstract

In the relatively short history of anti-tumor treatment, numerous medications have been developed against a variety of targets. Intriguingly, although many anti-tumor strategies have failed in their clinical trials, metformin, an anti-diabetic medication, demonstrated anti-tumor effects in observational studies and even showed its synergistic potential with immune checkpoint inhibitors (ICIs) in subsequent clinical studies. Looking back from bedside-to-bench, it may not be surprising that the anti-tumor effect of metformin derives largely from its ability to rewire aberrant metabolic pathways within the tumor microenvironment. As one of the most promising breakthroughs in oncology, ICIs were also found to exert their immune-stimulatory effects at least partly via rewiring metabolic pathways. These findings underscore the importance of correcting metabolic pathways to achieve sufficient anti-tumor immunity. Herein, we start by introducing the tumor microenvironment, and then we review the implications of metabolic syndrome and treatments for targeting metabolic pathways in anti-tumor therapies. We further summarize the close associations of certain aberrant metabolic pathways with impaired anti-tumor immunity and introduce the therapeutic effects of targeting these routes. Lastly, we go through the metabolic effects of ICIs and conclude an overall direction to manipulate metabolic pathways in favor of anti-tumor responses.

## 1. The Tumor Microenvironment and Immune Checkpoint Inhibitors

**Figure 1:**
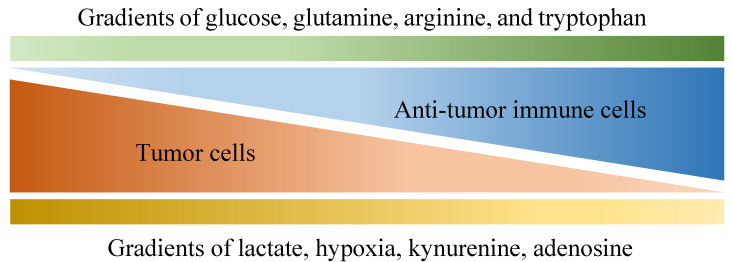
Various gradients within the tumor microenvironment that are differentially associated with anti-tumor activity and tumor growth. Tumor cells are known for their aberrant metabolic activity that leads to local depletion of a variety of nutrients, including glucose, glutamine, arginine and tryptophan, which effectively hinder anti-tumor activities provided by immune cells that also depend on these nutrients. In addition, metabolites such as lactate, kynurenine and adenosine are released by tumor cells, dampening anti-tumor immunity along with hypoxia.

Of all diseases, cancer has long been one of the major health concerns in spite of extensive research exploring ways to tackle it. Due to its abnormal growth and deadly metastasis irrespective of limited oxygen and nutrients, the deregulating cellular energetics and the immunoevasion nature of cancer have been identified, which are largely derived from its ability to recruit normal cells that constitutively create the “tumor microenvironment” (TME) [1]. The TME is characterized by a complex interplay between tumor cells and their surrounding neighbors, including stromal cells, extracellular matrix, adipocytes, mesenchymal stem cells, blood vessels, macrophages, T cells, B cells, cytokines, exosomes and metabolites. All the components within the TME contribute to building a 3-dimensional structure with gradients of oxygen tension and availability to nutrients (as shown in Figure 1) [2,3], which favors the development of tumors in multiple aspects such as local progression and distal metastasis [4,5,6,7,8,9]. Importantly, although immune cells are known to protect us from tumors through their immunosurveillance and tumoricidal nature [10,11], multiple factors within the TME may not only hinder their antitumor function but also skew them to construct an immunosuppressive environment in favor of tumor growth.

In the devastating battlefield against tumors, a variety of protumoral factors and immunosuppressive mechanisms have been identified, of which the immunosuppressive cells, exosomes and the co-inhibitory signals play central roles to allow tumor progression. To begin with, the immunomodulatory cells, including but not limited to regulatory T (Treg) cells and M2 macrophages, accumulate in the TME and diminish T cell anti-tumor immune responses [12,13]. Treg cells are famous for their immunosuppressive effects on not only aberrant immune responses against self-antigens but also anti-tumor immune responses, in both laboratory and clinical studies [14,15,16]. Treg cells are believed to modulate immune responses through expressing immunosuppressive cytokines (including transforming growth factor (TGF)-β, interleukin (IL)-10, IL-35), immune checkpoints (such as cytotoxic T-lymphocyte-associated antigen (CTLA)-4; programmed death (PD)-1) and other co-inhibitory receptors, as extensively reviewed by previous studies [17,18]. Furthermore, Treg cells are also capable of inducing tolerogenic dendritic cells (DCs) that are linked to T cell exhaustion, and can release cytotoxic agents such as perforin and granzyme [19,20,21,22,23]. Therefore, the aberrant accumulation of Treg cells in TME impairs anti-tumor immunity through various mechanisms [24,25], facilitating tumor growth and progression. Besides Treg cells, the unwanted immunosuppression within the TME can also be affected by tumor-associated macrophages (TAMs) that are majorly driven by cytokines such as IL-4 or IL-13, which have also earned their “M2-like” naming [26,27,28]. Orchestrated by signals from tumor cells, T cells as well as stroma [29,30], TAMs play a protumoral role in the TME by promoting tumor metastasis through promoting angiogenesis as well as extracellular matrix remodeling [31,32]. Moreover, TAMs also exert their profound immunosuppressive effects through expressing a variety of inhibitory ligands and cytokines, including PD-L1, PD-L2, B7-1, B7-2, HLA-G, HLA-E, IL-10 as well as TGF-β, as clearly reviewed by previous studies [33,34]. Besides immunosuppressive cells, previous studies further reveal the vital roles of exosomes and co-inhibitory molecules to promote tumor growth and metastasis in many different ways. 

The aberrant accumulation of immunosuppressive cells within the TME is thought to be affected by both exosomes and co-inhibitory signals derived from the tumor cells. Exosomes are extracellular vesicles with bi-layered membrane that range between 30-100 nm in diameter [35], while patients with cancer, especially of the ones with poor prognosis, are often found with higher numbers of them [36]. Tumor-derived exosomes have been detected in many different types of cancers, and they can not only impair anti-tumor immunity within the TME but also educate bone marrow-derived progenitor cells to facilitate distant metastasis [37,38,39,40]. On the other hand, the expression of co-inhibitory molecules such as PD-L1 have been observed on various kinds of cancer cells [41], which correlates with poor clinical outcomes of many patients with cancer [42,43]. Importantly, since the co-inhibitory checkpoint molecules expressed by both tumors and immunosuppressive cells within the TME dampens anti-tumor immunity of T cells [44,45,46,47], immune checkpoint inhibitors (ICIs) such as the antibodies directed against CTLA-4, PD-1 and PD-L1, are developed into encouraging treatments against various tumors, most notably melanoma and non-small cell lung cancer [48,49,50,51]. For instance, a pooled meta-analysis assessing long-term survival of 1861 advanced melanoma patients estimated a 3-year survival rate of 22% for patients receiving anti-CTLA4 ipilimumab [52], which evidently outperformed other chemotherapy such as dacarbazine, where the 3-year survival rates were only around 12% [53]. However, despite the game-changing efficacies of ICIs against tumors in clinical scenarios, they still face improvable downsides, including the suboptimal long-term response rates due to both innate and acquired resistance [54], as well as the lack of a reliable predictive biomarker [55]. Therefore, a variety of combinatory therapeutic strategies, along with the discovery of potential biomarkers, have been tested out to overcome the limitations, and targeting the metabolic pathways within the TME has appeared as one of the emerging candidates that have synergistic potential.

## 2. The Implications of Metabolic Syndrome for Targeting Metabolic Pathways within the Tumor Microenvironment

When it comes to the phrase “metabolic”, metabolic syndrome pops up in many physicians’ minds due to its extremely high prevalence as well as its modifiable nature [56,57,58]. As a cluster of unfavorable conditions including increased blood pressure, blood sugar, cholesterol levels, and body fat around the waist, the poor control of metabolic syndrome is widely believed to result in metabolic diseases such as type 2 diabetes mellitus [59,60,61], which is characterized by hyperglycemia and hyperinsulinemia due to increased insulin resistance. Although these pathological conditions are believed to lead to further complications such as stroke, heart attack, diabetic foot and diabetic nephropathy, accumulating epidemiological evidence also confirms their close relationships with not only worse prognosis in tumor patients but also an increased risk for tumor [62,63,64,65,66,67,68]. For instance, a meta-analysis study sums up the epidemiological evidence, indicating that the incidences of various cancers, including pancreas, liver, breast, colorectal, urinary tract and female reproductive organs, are increased in diabetic patients [69]. Furthermore, a recent study has estimated that of the documented global tumor cases in 2012, 2.0% of them were attributable to diabetes mellitus alone, whereas 5.6% of them were attributable to the combined effects of both diabetes and high BMI [64]. Although systemic manifestations such as hyperglycemia, insulin resistance, hyperinsulinemia and chronic inflammation have provided clues to the origin of these associations [70,71,72,73,74,75,76,77], it is difficult to delineate the underlying mechanisms based on macro-level perspectives without knowing how each pathological molecule affects the pathogenesis. Therefore, physicians must look into the subcellular and molecular course of events, such as the metabolic pathways.

Metabolic pathways are the consecutive series of biochemical reactions that take place in a single cell, enabling a variety of cellular functions. Besides the discussed factors that favor tumor growth within the TME, it is also indispensable to keep an eye on the related metabolic pathways, especially of the one involving the insulin-like growth factor (IGF) system, to elucidate the underlying connection between metabolic syndrome and tumor [78]. The IGF system consists of three molecules that induce their cellular responses mainly through IGF-1 receptor (IGF-1R), namely insulin, IGF-1 and IGF-2 [79]. IGF-1 is a 70-aminoacid polypeptide hormone mainly produced by the liver [80], and it shares structural homology with IGF-2 and proinsulin [81]. IGF-1 can either bind to its putative receptor (IGF-1R) or the insulin receptor, while there is also a hybrid form of receptor which both insulin and IGF-1 can ligate to [82,83]. After binding, these receptors elicit downstream tyrosine kinase activity and activate the Ras-mitogen-activated protein kinase and phosphatidylinositol 3-kinase (PI3K)/Akt pathways [84,85]. Whilst the Ras-mitogen-activated protein kinase mediates the mitogenic effect, the PI3K/Akt pathway activates enzymes involved in gluconeogenesis, glucose uptake, protein synthesis and lipogenesis, reprogramming the metabolic pathways [79,86]. In tumor cells, the IGF-1 signaling drives neoplastic behaviors [87,88,89], as well as protects tumors from cytotoxic treatment likely through promoting the activity of efflux pumps while upregulating double-strand break repair [90,91,92]. The clinical relevance of these in vitro findings was confirmed by, for example, an immune-histochemical analysis of pancreatic cancer biopsies that identified activated insulin/IGF receptors expressed in 72% of the patients, which positively correlates with the infiltration of TAMs that secrete IGF-1 and IGF-2 along with tumor stromal myofibroblasts, supporting the chemo-resistance of the tumors [93]. Furthermore, the insulin receptor was found to be overexpressed by the angiogenic vasculature in human tumors, facilitating tumor angiogenic activity and correlates to shorter survival [94]. In addition, hyperinsulinemia also promotes carcinogenesis indirectly by reducing the hepatic production of IGF binding proteins [95,96,97], thereby elevating the circulating levels of bioactive IGF-I and, therefore, further fuels the growth of neoplastic cells [78,98]. On the other hand, although the endocrine-immune axis of the IGF system is much more complicated [99,100], evidence has suggested its overall immunosuppressive effects by increasing the infiltration of Treg cells in affected tissues [101,102,103], enhancing the expression of immunosuppressive cytokines as well as inducing the M2 phenotype of macrophages that secretes anti-inflammatory cytokines [104,105,106,107]. In short, the excessive insulin in patients with metabolic syndromes facilitates these metabolic pathways in the TME, which potentiates tumor growth and compromises antitumor activity. After reviewing the basic findings in metabolic pathways, it is, therefore, unsurprising for physicians to observe that tumor risk is higher [108], as well as that the prognosis of some tumors is worse in patients with hyperinsulinemia, shedding light on the therapeutic potentials of targeting these metabolic pathways [72,109]. 

Before developing targeted therapy against the aberrant IGF system within the TME, clinical observations had revealed the close relationship between certain anti-diabetic strategies and their protective effects against cancer. To begin with, losing weight is known to improve not only the outcome of type 2 diabetes through enhancing overall glycemic and metabolic control [110,111], but also provide anti-tumor benefits in clinical scenarios [112,113,114]. This approach increases serum IGFBP-2 level and decreases the number of infiltrating macrophages in both adipose tissue and tumors of colon and liver [115,116,117], likely improving the prognosis through reducing the metabolic signals originated from the IGF system. Second, as the long been preferred first-line oral anti-diabetic agent to manage type 2 diabetes [118], Metformin exerts its anti-diabetic effects through decreasing hyperinsulinemia and insulin resistance [119,120], but it is also known of its anti-tumor activity. In vitro studies of Metformin showed that it inhibits tumor cell proliferation through partial cell cycle arrest [121,122,123] and activates 5′ adenosine monophosphate-activated protein-activated protein kinase in tumor cells, leading to growth inhibition by inhibiting protein synthesis [124]. Furthermore, in vivo studies have pointed out that metformin has more anti-tumor activities in mice receiving a high-energy diet, associated with hyperinsulinemia and accelerated tumor growth, than it does in mice receiving a balanced diet [125], indicating that the insulin-lowering effect of metformin likely contributes to its anti-tumor activity [126,127]. Besides, clinical evidence also suggests that elevated insulin and IGF-1 levels favor the progression of a variety of cancers [128,129]. Therefore, it is not surprising that a substantial number of observational human studies suggest that treatment with metformin reduces cancer risk as well as cancer mortality [130,131,132,133,134,135], which is in clear contrast to the increased risk for cancer or cancer death associated with the use of exogenous insulin, insulin analog glargine or sulfonylureas [136,137,138]. For instance, a systemic review and meta-analysis have noted a 31% reduction in overall summary relative risk for cancer in patients taking metformin compared with other antidiabetic medications and a trend to a dose–response association was also found [139]. In contrary, it was reported that insulin glargine, which would be ultimately required by many diabetic patients [140], has elevated mitogenic potency [89,141,142], supporting the aforementioned clinical findings as well as the importance of this metabolic pathway in oncology. Although confounding by indication from observational studies may affect the interpretation of results, experimental studies have additionally supported these findings by revealing the metformin-induced energy stress on tumor cells that reduce their mitochondrial ATP output, turning them into a more glycolysis-dependent state [143,144], especially in tumors lacking LKB1 or p53 [126,145]. In short, it is evident that the aberrant IGF system contributes, at least partly, to the tumor development and growth, so agents that target this metabolic axis have been tested out.

Several inhibitors of the IGF system have been developed as anti-tumor treatments, including small molecules that inhibit PI3K or its downstream signal Akt [146,147]. In addition, medications such as sorafenib have also demonstrated ability to reduce the expression of IGF-1 by M2 macrophages as well as the following IGF-1-driven hepatocellular carcinoma growth in a preclinical study [148]. Although the rationale of targeting this pathway as well as its preclinical studies seem promising, the single-agent strategy has yet to find great success in clinical trials [149,150,151]. Besides the undesired inhibition of insulin action in the liver, muscle and fat that results in metabolic toxicity and likely precludes their use at clinically effective doses [152], monotherapy against the IGF system may also downregulate anti-tumor immunity. For instance, it was demonstrated that the silencing of the insulin receptor reduced glucose transport and glycolysis in activated CD4+ T cells, resulting in enhanced apoptosis [153]. Furthermore, knockdown of the insulin receptor also reduces cytotoxic responses and proliferation rates of CD8+ T cells, while leaving the immunosuppressive Treg cells unaffected [153]. Taking into account the downsides of targeting this pathway alone, various combinatory therapies have been investigated. For instance, in vivo studies have noted that the inhibition of IGF-1/IGF-2 sensitized pancreatic tumors to gemcitabine [93], whereas combined IGF-1R and CSF-1R inhibition significantly improved survival outcomes in mice with glioblastoma multiforme [154]. Furthermore, since downregulating IGF-1 or IGF-1R has been found to enhance the immunogenicity of GBM models in rats and a small clinical study in astrocytoma [155,156,157], possibly partly contributed by inducing tumor cell death to release tumor-specific antigens [158], further investigation of the anti-tumor efficacy of combining ICIs with anti-IGF strategies is also warranted.

Currently, although there may be inadequate evidence to evaluate the effect of combining ICIs with anti-IGF treatment against tumors, the combination of ICIs with metformin did show some encouraging results. A retrospective cohort study of patients with metastatic malignant melanoma revealed favorable treatment-related outcomes in terms of objective response rate, disease control rate, overall survival and progression-free survival in patients who received metformin in combination with ICIs, as compared to patients receiving ICIs alone [159]. Another retrospective descriptive analysis of the randomized phase III OAK trial for the treatment of advanced or metastatic previously treated non-small cell lung cancer also demonstrated a synergistic benefit of metformin treatment in addition to the anti-PD-L1 antibody atezolizumab in the overall response rate [160]. The observed anti-tumor potential of metformin may not only derive from downregulating the IGF system by decreasing hyperinsulinemia but also contributed by regulating immunometabolisms [161]. By blocking mTOR signaling and restoring mitochondrial fatty acid oxidation, metformin facilitates the shift of T cell metabolism from a glucose-dependent anabolic state to a catabolic state of metabolism, which resembles memory T-cell [162,163]. Therefore, it may not be surprising that a clinical trial of head and neck squamous cell carcinoma showed that metformin use was linked to a 66.5% increase in CD8+ effector T cells and a 41.4% decrease in FoxP3+ Treg cells within the TME [164]. Although metformin is likely to work synergistically with ICIs by modulating metabolic pathways of tumor cells and immune cells, the observed benefits may also contribute to its ability to not only inhibit the tolerogenic M2-TAMs [165], plausibly via modifying their lipid metabolism and kynurenine metabolic pathway [166,167], but also enhance PD-L1 degradation [168]. In light of the possible therapeutic mechanisms, it is also worth noting that these combinatory treatments may identify suitable patient populations and further optimize treatment outcomes by developing predictive biomarkers (e.g., hyperinsulinemia) based on the information.

## 3. The Metabolic Pathways in the Tumor Microenvironment: Glucose and Lipid

Besides the aforementioned metformin and IGF system, many other developing strategies aim to direct metabolic pathways of immune cells and/or tumor cells to overcome the immunosuppressive environment within the TME. As introduced in the previous sections, despite a variety of immune cells being found within the TME, such as DCs, macrophages, T cells, as well as B cells, plasma cells and so on, they either educate each other through anti-inflammatory signals to construct an immunosuppressive condition or face a hostile environment consisting of nutrient scarcity, metabolite abundance and hypoxia [169]. Cytotoxic CD8+ T lymphocytes are known to be the main antitumor effector cells [170,171] which initiate their tumoricidal function after priming by antigen-presenting cells (APCs) such as macrophages and DCs [172,173]. However, although TAMs are considered as the most abundant APCs within the TME, they often have poor antigen-presenting capacity [172,174]. On the other hand, the priming process can neither rely on DCs, since both intratumoral and systemic DCs are found to be dysfunctional and/or reduced [175,176,177]. The dysfunctional, or even skewed, anti-tumor activities were considerably affected by the altered metabolic pathways, which are turning out to be one of the most promising anti-tumor targets.

In fact, cancers favor glycolysis for energy even in the presence of sufficient oxygen, known as the Warburg effect, which forms the basis for diagnostic tools like 18F-fluorodeoxyglucose-positron emission tomography [178]. The net gain of anaerobic oxidation of 1 mole of glucose is 2 moles of ATP, which is far less than the following oxidative phosphorylation that generates 36 additional moles of ATP, this means a much higher amount of glucose consumed for energy maintenance under anaerobic conditions. Since glycolysis is also required by effector T cells [179,180,181,182], as well as activated macrophages and DCs [183,184], the competitive TME impairs T cells by limiting their mTOR activity as well as glycolytic capacity [185,186], while interrupting the metabolic programs and promoting the development of tolerogenic DCs and M2-like macrophages [187,188,189]. Furthermore, besides reprograming Th17 cells into Treg cells [180], limited glycolysis also inhibits the expression of interferon-γ by Th1 cells [190,191] and hinders the anti-tumor effector functions of Th1 CD4+ T cells while upregulating TGF-β production [186]. Interestingly, it was reported that inhibited glycolysis during CD8+ T cell differentiation skews effector T cell generation towards a more central memory phenotype [192,193]. On the other hand, the TCA cycle and oxidative phosphorylation are associated with a more anti-inflammatory phenotype [194,195,196,197] and are prominent in memory CD8+ T cells [198]. In contrast to glycolysis, generating energy through fatty acid oxidation (FAO) has been observed in many non-inflammatory immune cells with increased cellular lifespans, including M2 macrophages, Treg cells and memory T cells [199]. FAO promotes the generation of Treg cells while inhibiting effector T cell polarization in favor of tumor growth [179]. However, FAO also plays a key role in the generation and maintenance of memory CD8+ T cells, which is being acknowledged as the main contributor of long-lived immunity and activated DCs against tumors [200,201]. Memory CD8+ T cells appear to rely on FAO to have timely responses to antigen stimulation [202], while treatment like IL-15 promotes their FAO and results in increased cell survival [203]. In addition, since targeting lipid synthesis in tumor cells inhibits tumor progression [204,205,206], whether it also affects the immune system has been evaluated. It was found that increased fatty acid synthesis (FAS) is necessary for DC activation and their subsequent stimulation of CD8+ T cells [207]. FAS is also important for both T cells and B cells, especially for cell proliferation after activation [208,209]. Intriguingly, T cell-specific deletion of acetyl-CoA carboxylase 1 (ACC1), the rate-limiting enzyme in FAS, was found to reduce blasting efficacy along with a lower accumulation of antigen-specific CD8+ T cells, which could be reversed by supplementing them with exogenous fatty acids [210]. However, neither pharmacological nor genetic inhibition of ACC1 in CD4+ T cell subsets seems to affect Treg cell generation and function [211]. Due to the multifaceted influences of targeting metabolic pathways for anti-tumor therapy, there could plausibly be inefficacies in their monotherapy, which are expected to be overcome by combinatory treatments.

Since the TME is depleted of glucose [212], it inhibits CD4+ effector T cells and promotes Treg cells, generating an immunosuppressive environment. Therefore, several strategies have been under investigation to rewire the aberrant metabolic reprogramming. Enhancing memory-associated metabolism, including oxidative phosphorylation and FAO, by small-molecule inhibitors which can enforce mitochondrial fusion or inhibit mitochondrial fission, generated CD8+ T cells and controlled tumor growth in vivo [213]. Furthermore, several studies have demonstrated that the co-stimulatory receptor 4-1BB is key to maintaining mitochondrial health and biogenesis for robust anti-tumor immunity [214,215]. Similarly, metabolic interventions can also be directed against tumor cells, as inhibiting tumor glycolysis also improves T cell-mediated anti-tumor effects both in vitro and in vivo [216], while another study has shown that the efficacy of ICI therapy was promoted by the inhibitory effect of metformin on oxygen consumption of tumor cells, thereby reducing intra-tumoral hypoxia and potentiating anti-tumor immunity [217]. In short, treatments that open a door to rewire the TME-associated aberrant metabolic landscape concerning insufficient glycolysis accompanied by different degrees of hypoxia hold the potential to aid in future anti-tumor strategies.

When it comes to improving treatments against tumors by manipulating metabolisms of fatty acids, targeting FAO, preferentially utilized by non-inflammatory and tolerogenic immune cells and FAS, resembled inflammatory responses, have gradually discovered encouraging findings [199]. It is known that regulating cholesterol metabolism in CD8+ T cells is important to maintain a strong TCR clustering during activation [218]. As a result, Avasimibe, an inhibitor of the cholesterol esterification enzyme acetyl-CoA acetyltransferase (ACAT) 1, was developed, demonstrating enhanced proliferation as well as effector function of CD8+ T cells [218]. In addition, TVB-2640, an inhibitor of FAS, had entered early clinical trials in light of the critical role that FAS plays in early T cell activation and clonal expansion, demonstrating prolonged stable disease across multiple tumor types [219]. Taken together, these studies point out the anti-tumor potential of targeting lipid metabolisms, which may work synergistically with the current state of ICIs.

## 4. The Metabolic Pathways in the Tumor Microenvironment: Amino Acids and Metabolic Intermediates

Effector T cells consume large amounts of glucose, the carbons of which are largely secreted as lactate [220], whereas amino acids serve as building blocks to support protein and nucleotide synthesis, which are especially required upon T cell activation [221]. To begin with, the important role for glutamine to fuel the TCA cycle and generate metabolic intermediates for anabolic proliferation has been well-documented in both cancer and immune cells [221,222]. It was noted that there is a marked increase in glutamine usage upon both T cell and B cell activation in order to respond to antigen stimulation [222,223,224]. Glutamine metabolism is also suggested to regulate the balance between effector T cells and Treg cells, because the impaired uptake of neutral amino acids caused by the genetic loss of alanine serine cysteine transporter 2 (ASCT2) in T cells results in hindered generation and function of Th1 and Th17 cells, while not affecting Treg cell generation [225,226]. Furthermore, both the reduction of glutamine levels in culture media and ASCT2 loss reduced mTORC1 activity, which coincided with the effector T cell defects [225]. Therefore, although glutamine antagonists such as 6-diazo-5-oxo-L-norleucine (DON) have the ability to inhibit tumor growth in clinical scenarios [227], likely thereby improving overall anti-tumor immunity, whether they would also hinder anti-tumor immunity metabolically remains controversial. DON is a glutamine analogue that can bind to the catalytic centers of various glutamine-utilizing enzymes such as GLS1 (glutaminase1), achieving glutamine blockade of many metabolic pathways [228,229]. Recently, the anti-tumor effects of glutamine blockade with the prodrug of DON, JHU-083, has been tested out in tumor-bearing mice [230]. It was found that glutamine blockade not only decreased hypoxia, acidosis and nutrient depletion within the TME by suppressing oxidative and glycolytic metabolism of cancer cells, but also markedly upregulated oxidative metabolism of CD8+ effector T cells, adopting a long-lived and highly activated phenotype. These studies proved to be highly instructive in terms of better understanding the differences in T cell and cancer metabolism as well as novel concepts concerning the metabolism of anti-tumor specific T cells. Indeed, glutamine helps to support massive clonal expansion of T cells and blocking glutamine pathways can limit the magnitude of clonal expansion. However, recall that after peak expansion there is significant contraction characterized by cell death. It was found that while inhibiting glutamine metabolism does limit peak expansion, it also reprograms the T cell metabolically such that they are more robust and do not undergo massive contraction. In the tumor setting, blocking glutamine metabolism markedly inhibits tumor growth while simultaneously reprogramming anti-tumor T cells to be more robust memory and effector cells. Additionally, glutamine metabolism also plays a critical role in M2 macrophage polarization by fueling the TCA cycle [194], where inhibition of glutamine synthetase skews M2 macrophages to an M1-like phenotype and prevents tumor metastasis [231]. Likewise, it was found that inhibition of glutamine metabolism employing the DON prodrug JHU-083 can inhibit MDSC generation, inhibit metastasis and promote anti-tumor immunity [232]. As a result, multiple clinical trials are being carried out to evaluate the safety and efficacy of glutaminase inhibitors in treating different tumors [233,234,235], and a combinatory approach for targeting this metabolic pathway is expected to lead to more promising results.

In addition, there has been much focus on the immune regulatory role of tryptophan metabolism particularly on indoleamine-2, 3-dioxygenase (IDO). The proliferation of T cells requires tryptophan [236], whereas driving IDO expression and subsequent tryptophan catabolism in APCs blunts T cell stimulation [237]. Indeed, tryptophan insufficiency can induce accumulation of charged tRNAs, while activating the unfolded protein response protein GCN2 [238]. Importantly, many other aspects of tryptophan metabolism, especially metabolites generated from tryptophan catabolism such as kynurenine, can modulate immune cell function through activation of the aryl hydrocarbon receptor [239]. IDO expression is generally seen in tumor cells, surrounding stromal cells and even TAM [240,241,242], while increased IDO expression correlates with a poor prognosis in certain cancers [243,244], likely through impairing the anti-tumor effects of T cells [245,246]. Although inhibition of IDO in murine tumor models stimulated the T cell-mediated anti-tumor responses [247,248], a recent phase III study of the direct IDO inhibitor, epacadostat, failed to further increase the progression-free survival in combination with anti-PD-1 therapy over anti-PD-1 monotherapy [249]. Therefore, alternative methods of inhibiting this pathway are being pursued, and, hopefully, may yield further clinical implications upon combining with anti-tumor agents.

Serving as a precursor for the biosynthesis of proteins as well as creatine, arginine is also required for effective immune responses. It was shown that exogenous l-arginine leads to improved T cell fitness as well as enhanced generation of central memory cells [250]. Arginine has also been noted to regulate the T cell receptor [251], as well as to support the proliferation of human T cells [252]. In addition, an adequate supply of arginine is also critical for M1 macrophages to generate nitric oxide for anti-microbial and cytotoxic functions [253]. However, the arginase expression in macrophages has been reported to limit the inflammatory response of effector T cells [254], possibly through suppressing mTORC1 activity due to arginine-depletion that has also been observed in glutamine deficiency [255,256]. This competition over arginine has been observed in various human cancers and mouse cancer models, where TAMs are identified to secrete arginase into the TME [257,258], metabolizing l-arginine and inhibiting T cells [259]. Based on this rationale, CB-1158, a potent inhibitor of arginase, has been under investigation as a strategy to shift the TME toward a pro-inflammatory environment [260]. So far this approach has shown good tolerance along with on-target inhibition [261], and even synergy with ICIs such as pembrolizumab [262].

Excessive amounts of the metabolites lactate and adenosine found in the TME serve to inhibit anti-tumor immunity. Many cancers rely on aerobic glycolysis, which produces a large amount of lactate and acidifies the TME, helping tumor growth while evading the anti-tumor responses [216,263,264]. Lactate can suppress immune responses against tumor by driving M2-like macrophage polarization [189,265], inhibiting NK cell activity [266], conversely favoring Treg cells [267], as well as blocking proliferation and functions of T cells [268,269,270]. As a result, there has been much interest in targeting l-lactate dehydrogenase (LDH) A chain, which catalyzes the formation of lactic acid [271]. Nevertheless, it is important to point out that effector T cells also rely on the activity of LDH for effective proliferative and functional responses [272,273]. Again, monotherapy that blocks LDH may, therefore, have unwanted effects in addition to the effector arm of the anti-tumor response [274]. Besides lactate, the concentration of adenosine is also markedly increased in hypoxic tissues and the TME [275], supporting angiogenesis and profoundly suppressing immunity [276,277]. Both CD39 and CD73 are cell surface molecules with critical roles in controlling the production of adenosine [278] and their upregulations in tumors are linked to poor prognosis [279,280,281]. Furthermore, Treg cells can also express CD39, causing immunosuppression as well as the abrogation of NK cell function in the TME [282,283]. Besides, adenosine also inhibits T cells from expressing the IL-2 receptor and TCR-stimulated proliferation [284], while upregulating the expression of immune-checkpoint receptors (including PD-1 and CTLA-4) [285]. The generally immunosuppressive effects discovered of this pathway paved the way for many pre-clinical studies, showing that targeting the adenosine-related pathways had synergistic anti-tumor effects along with ICIs [286,287,288]. An early clinical trial revealed that the use of CPI-144, an adenosine A2a receptor inhibitor, was associated with increasing disease control over refractory renal cell carcinoma and also acts synergistically with ICI [289], and there are currently a number of ongoing clinical trials [290].

To sum up, amino acids, along with lactate, generally have diverse immunomodulatory effects, which possibly require cell-specific manipulations or combinatory strategies to specifically rewire the metabolic pathways needed to maximize the efficacy of anti-tumor activity. On the other hand, targeting metabolites such as adenosine have yielded promising synergistic effects with ICIs so far, but further investigation is warranted.

## 5. The Effects of Exosomes and Immune Checkpoint Inhibitors on Metabolic Pathways

Since the immunosuppressive nature of the TME may largely be affected by both exosomes and co-inhibitory molecules, as discussed previously, looking into their effects on metabolic pathways is important for developing novel therapeutic strategies. To begin with, the acidified environment built up by lactate facilitates both the release and uptake of exosomes [291]. It was reported that gastric cancer-derived exosomes could transmit autocrine signals that promote tumor proliferation via increasing the phosphorylation of Akt [292]. Furthermore, as one of the major cell types within the TME, cancer-associated fibroblasts have been shown to produce exosomes that inhibit oxidative phosphorylation and increase glycolysis as well as glutamine-dependent reductive carboxylation in tumor cells [293]. In addition, these exosomes could also fuel nutrient-deprived tumor cells with TCA intermediates and glutamine [294]. Similarly, exosomes secreted by adipocytes have also been found to transmit proteins implicated in FAO that promote tumor migration and invasion [295]. Despite these intriguing findings, whether they could be further developed into successful treatment in clinical scenarios requires further research.

Given the therapeutic efficacy of ICIs in many cancers, it may not be surprising to find that they also influence metabolic programming as well as the nutrient competition between tumor cells and T cells. Interaction of PD-1 with its ligands promotes FAO [296], but prevents metabolic reprogramming including upregulation of aerobic glycolysis as well as glutaminolysis, in T cells through inhibiting the PI3K–Akt–mTOR pathway [297]. Interestingly, PD-L1 has also been found to affect the metabolism of tumor cells by stimulating aerobic glycolysis through activating the PI3K–Akt–mTOR pathway [185]. As a result, by inhibiting the PD-1–PD-L1 axis, a profound anti-tumor effect could be expected by restoring the metabolic fitness of T cells while downregulating aerobic glycolysis in tumor cells, thereby also increasing the availability of glucose to the T cells [216]. In addition to the PD-1–PD-L1 axis, the CD28-CTLA-4 axis also affects metabolic pathways. It is known that reducing Akt phosphorylation and activation contributes to the inhibitory effect of CTLA4 signaling on CD28-mediated co-stimulation [298], which possibly impairs the increased glucose metabolism and mitochondrial remodeling following T cell activation. However, unlike PD-1, CTLA-4 signaling does not seem to promote FAO [296]. To sum up, ICIs elicit profound anti-tumor activities at least partly affected by their ability to modify metabolic pathways and win the tug-of-war over nutrients in TME, suggesting the importance to incorporate thoughts on metabolic pathways when designing anti-tumor strategies. It is vital to keep in mind, however, that not only do different immune cells depend on different metabolic pathways (as shown in Table 1) but also there are likely to be numerous mechanisms through which each metabolic pathway acts to modulate the immune system. Future work on further elucidating the metabolic effects of various anti-tumor agents on both immune and tumor cells could yield new insights to treat various cancers.

## 6. Conclusions

In the history of oncology, there have already been numerous strategies against cancers proposed, but only a small proportion of them have proved to be effective in clinical trials. Intriguingly, anti-tumor effects were found not only in the successful development of ICIs but also in various drugs which target metabolism. Looking back from bedside-to-bench, the metabolic pathways within the TME appear to be increasingly important in oncology. There is a strong rationale supporting the combination of metabolic interventions with many other developed therapies against cancer, and a variety of ongoing clinical trials are being carried out [299]. Targeting the metabolic pathways is undoubtedly important in breaching the immunosuppression imposed by the TME, and potentially even directly improving anti-tumor immunity by targeting suppressive cells like Tregs and MDSC, enhancing the generation of memory cells and effector functions.

## Figures and Tables

**Table 1:** The role of different metabolic pathways in different kinds of T cells. Increased glycolysis and fatty acid synthesis is required for proliferation and differentiation of effector T cells upon activation. On the other hand, fatty acid oxidation is important for the development of CD8+ T cell memory and for the differentiation of CD4+ regulatory T cells as well. OXPHOS, oxidative phosphorylation; FAO, fatty acid oxidation; FAS, fatty acid synthesis.

	Glycolysis	OXPHOS	FAO	FAS
Naïve T cell		○	○	
Effector T cell	○	○		○
Memory T cell		○	○	○
Regulatory T cell		○	○	

## Abbreviations

TME	tumor microenvironment
Treg	regulatory T
TGF	transforming growth factor
IL	interleukin
CTLA-4	cytotoxic T-lymphocyte-associated antigen 4
PD-1	programmed death 1
DC	dendritic cell
TAM	tumor-associated macrophage
ICI	immune checkpoint inhibitor
IGF	insulin-like growth factor
PI3K	phosphatidylinositol 3-kinase
APC	antigen-presenting cell
FAO	fatty acid oxidation
FAS	fatty acid synthesis
ACC1	acetyl-CoA carboxylase 1
ACAT	acetyl-CoA acetyltransferase
ASCT	alanine serine cysteine transporter
DON	6-diazo-5-oxo-L-norleucine
IDO	indoleamine-2,3-dioxygenase
LDH	lactate dehydrogenase
OXPHOS	oxidative phosphorylation
